# Perceived quality of life and associated factors among patients with severe mental illness in Ethiopia: a cross-sectional study

**DOI:** 10.1186/s40359-021-00664-w

**Published:** 2021-10-04

**Authors:** Seid Shumye, Tadele Amare, Habtamu Derajew, Merdia Endris, Wondwosen Molla, Nebiyu Mengistu

**Affiliations:** 1 Department of Psychiatry, Dilla University, P.O. Box (DU): 419, Dilla, Ethiopia; 2 Department of Psychiatry, University of Gondar, Gondar, Ethiopia; 3 Department of Psychiatry, Amanuel Mental Specialized Hospital, Addis Ababa, Ethiopia; 4 Department of Midwifery, Dilla University, P.O. Box (DU): 419, Dilla, Ethiopia

**Keywords:** Quality of life, Severe mental illness, Amanuel mental specialized hospital

## Abstract

**Background:**

Severe mental illness is strongly associated with an impaired quality of life. This intern can affect the treatment adherence and outcomes of the illness. However, there are insufficient studies in the literature pertaining to the quality of life of patients with severe mental illness in Ethiopia. Therefore, assessing the quality of life of patients with severe mental illness and its correlates is a yardstick measure of the effectiveness of the mental health service.

**Methods:**

An institutional based cross-sectional study was conducted from May 1 to 16, 2019 at Amanuel Mental Specialized Hospital. A systematic random sampling technique was used to get a total number of 387 samples. Data were collected using interview-administered questionnaires; World Health Organization Quality of Life Brief Version, Morisky Medication Adherence Screening Tool, Oslo Social Support Scale, and Jacoby Stigma Scale. Simple and multiple linear regression analysis were used to assess the contributing factors of quality of life in the participants and B coefficient with 95% CI confidence interval was used. The statistical significance was accepted at *p* value < 0.05.

**Results:**

The result showed that the Mean quality of life score of patients with severe mental illness for each domain (mean ± SD) was 41.3 ± 7.5, 42.8 ± 8.2, 38.9 ± 8.9, and 41.8 ± 6.5 for physical, psychological, social and environmental, respectively. Multiple regression analysis showed that age of participants was strongly positively correlated with all domains of quality of life. It predicts above 45% of the variability in each domain. Social support is also another strong predictor which was negatively correlated with all domains of quality of life, except physical.

**Conclusion:**

This study revealed that the mean score quality of life of patients with severe mental illness in each domain was low. This demonstrates a need for improving the quality of life of people with severe mental illness by integration of a positive mental health approach and bio-psychosocial view with biological treatment of severe mental illness. Moreover, in Collaboration with medical professionals, people with severe mental illness should screen and managed for any comorbid medical conditions.

## Background

Quality of life is defined by the World Health Organization as “individuals' observations of their position in life in the perspective of their culture and value systems and including the persons' physical health, psychological state, level of independence, social relationships, personal beliefs and their relations to outstanding features of the environment [1]. In recent times, quality of life is considered as an important indicator of the impact of diseases on the patients who are suffering from severe mental illness and is significantly affected in those patients [2].

Severe mental illness (SMI) is a name given for groups of mental health problems explained by a mental, behavioral, or emotional disturbances which markedly affects functionality, major life activities and quality of life of people suffering with the illness [3]. The World Health Organization estimated that severe mental illness (SMI) affects 4% of the adult populations worldwide [4], 4.2% in U.S adults [3], and 4.4% in Africa [5]. In Ethiopia, the prevalence of schizophrenia and bipolar disorder is estimated to be 4.8% and 2.0%, respectively [6].These disorders are stated as most important causes of disability due to health-related conditions representing a total of 19.1% in developing countries [7] and they are the major causes for social and functional impairment of people with SMI in Ethiopia [8]. People with severe mental illness have a diminishing QOL, frequently at levels that are equal to or exceed those of medical illnesses [9]. This significantly affects the treatment adherence, rates of relapse, ability to perform and/or enjoy occupational and social activities, future outlook, medical problems [2] [10–12]. Perceived stigma and drug non adherence were negatively affects the quality of life of patient with severe mental illness [13, 14].

Patients with severe mental illness and their relatives are increasingly in need of improvements not only their symptoms, but their functionality and quality of life of patients too [15]. This concludes that there should be a successful holistic approaches of treatment considering impairments in functionality and QOL [16, 17]. However, professionals have a considerable focus only on pharmacological treatments for symptomatic recovery by neglecting the other social, psychological, and environmental conditions of the client [9, 18].

Despite this diverse and complicated out comes, quality of life of people with severe mental illness is not well addressed in developing countries, particularly in Ethiopia. Therefore, this study was aimed to assess the quality of life and its correlates among people with severe mental illness in Ethiopia which will be used as a base line for further investigations.

## Materials and methods

### Study design and period

An institutional based cross sectional study was conducted from May 1 to 16, 2019 at Amanuel, Mental Specialized Hospital.

### Study setting

The study was conducted at outpatient clinic of Amanuel Mental Specialized Hospital. The hospital is the only specialized psychiatric hospital in the country since its establishment, 1930. It serves for patients coming from the entire regions of the nation. The hospital has 259 beds and 18 outpatient departments that serve patients with psychiatric disorders, of these 8 outpatient departments serve for patients with SMI. The hospital has served an average of 5,442 patients with severe mental illness are visiting the hospital monthly.

### Sample size and sampling

The number of sample required for the study was calculated using single population mean with assumptions of ^2^ = standard deviation of the mean quality of life score SD from a previous published study in Nigeria is 9.65 [19] with Margin of error (1 unit). By adding a 10% of non-response rate, the final sample size became 394. All patients with severe mental illness who were eligible for the study (age ≥ 18 years and received treatment for at least 6 months) were included.

Systematic random sampling was employed to select the study participants. Initially, the total expected number of patients with severe mental illness during the study period was calculated from the records of the hospital. Then, the sampling interval (K) was determined by dividing the total number of eligible individuals to the sample size to be drawn. Lottery method was used to select the first participant between one and K. Subsequently, K value was added until the proposed sample size was reached.

### Instrument

Person-Interviewed administered questionnaire was used for data collection. The questionnaire was included socio-demographic factors, clinical factors, psychosocial characteristics, and the World Health Organization Quality of Life–Brief (WHOQOL-BRFE).

The dependent variable was measured using the WHOQOL-BRFE. The measurement had 26 items measuring the physical health, psychological state, social relation, and environment perspectives. To measure the physical health domain of the WHOQOL-BRFE, sleep pattern, working capacities, energy and medications use items were used. Person’s thinking, body image and spiritual aspect items were used to assess the psychological state whereas the social domain was assessed by asking participants about their sexual relation and relationship with families. Moreover, environmental domain addressed the safety, leisure, finance, home and information aspects. This tool was cross-culturally validated instrument to measure the quality of life at health care settings with good sensitivity and specificity and in this study the Cronbach alpha was 0.88. The tool raw scores are transformed in to a range between 0 and 100 and they are scaled in a positive direction (i.e. higher scores related to a better health related quality of life and vice versa [20].

Factors associated with the outcome variable were assessed using standardized tools. Accordingly, the participant social support level was measured by Oslo social support scale (OSSS-3). OSSS-3 has been used for both epidemiological and population-based surveys. It has three items. The first item has four responses ranging from 1 (none) to 4 (more than five) while the second and third items have five choices. The total score of OSSS-3 ranges from 3 to 14. The higher score indicates the stronger levels of social support and vice versa. After the sum of total score, the social support level was categorized into three levels (poor = “3–8”, moderate = “9–11”, strong = “12–14”) [21]. In the current study, OSSS-3 showed a good internal consistency and had a Cronbach alpha of 0.93. Medication adherence level was measured by Morrisky Green Levine Medication Adherence Scale. This tool has four different “Yes” or “No” items scored for 1 and 0, respectively and individuals with a total score of four and above were considered as having poor medication adherence [22]. The Jacoby Stigma Scale with three item questions was used to assess the individual’s perception of stigma regarding their illness. Each of the three questions had two possible answers and scored 0 for “no” responses and 1 for “yes” responses.

To say the patient is stigmatized, the sum score should be 1 and above [23]. In the current study, the tool has a Cronbach alpha of 0.76. To measure current substance use, we directly asked the participants “Yes” or “No” question whether he/she had used substance in the last 1 month or not. The socio demographic factors (age, sex, religion, ethnicity, marital status, educational status, occupational status residency, and living arrangement) and clinical factors (age of onset of illness, type of diagnosis, duration of the illness and comorbid medical diagnosis) were assessed using Amharic Version of structured and semi structured interviewer administered questionnaire and chart review.

First, the questionnaire was prepared in English and translated into the local language (Amharic) and then back to English by senior English language expertise to check the accuracy. The questionnaire was pretested at St. Paul’s hospital among 5% of the calculated sample. Two days training were given for the data collectors and supervisors.

### Data analysis and interpretation

The collected data were coded, entered in to EPiDATA version 3.1 and exported to SPSS version 20 for analysis. Simple and multiple linear regression analysis were used to assess the correlates of independent factors with perceived quality of life as with a *p* value of < 0.25 were considered as candidates of multiple linear regressions. Variables with *p* value less than 0.05 were considered as significantly correlated with quality of life and B coefficient was used to predict the strength of the correlations of variables with quality of life.

## Results

### Socio-demographic and clinical characteristics of respondents

Out of 394 study participants invited for interview, 387(98%) participated in the study. The mean and standard deviation of age of respondents was 40 and (SD ± 8) years respectively. More than half, (58.4%) of respondents were males and 80.6% reside in urban areas. Majority (54%) of the participants married. Most, (61.0%) of the participants had a diagnosis of schizophrenia and 16% were bipolar disorder. The current use of substances among 387 study participants was 117(30.2%). Among those substance users, majority 47 (12.1%) use alcohol followed by 39 (10.07%) current khat/caffeinated drinks (Table 1).

**Table 1:** Socio-demographic and Clinical characteristics of patients with SMI having follow up at AMSH, Addis Ababa, Ethiopia, 2019 (n = 387)

Variables	Categories	Frequency	Percentage
Age (M ± SD) 40 ± 8			
Sex	Male	226	58.4
	Female	161	41.6
Ethnicity	Amhara	180	46.5
	Oromo	160	41.3
	Tigre	30	7.8
	Others*	17	4.4
Religion	Orthodox	201	51.9
	Muslim	103	26.6
	Protestant	70	18.1
	Others**	13	3.4
Residence	Urban	312	80.6
	Rural	75	19.4
Marital status	Single	209	54.0
	Married	99	25.6
	Divorced	60	15.5
	Widowed and separated	19	4.9
Educational status	Unable to read and write	24	6.2
	Primary education	110	28.4
	Secondary education	73	18.9
	Diploma, degree and above	180	46.5
Occupational status	Private business	141	36.4
	Unemployed	150	38.8
	Employed	79	20.4
	Others****	17	4.3
Living arrangement	With families	280	72.4
	Alone	107	27.6
Age of onset the illness(Mean ± SD) 31 ± 10			
Type of diagnosis	Schizophrenia	236	61.0
	Bipolar	62	16.0
	Major depression	89	23.0
Duration of the illness	Less than 5 years	100	25.8
	5–10 years	219	56.6
	Greater than 10 years	68	17.6
Comorbid medical diagnosis	No	320	82.7
	Yes	67	17.3
Medication adherence	Adherent	191	49.4
	Non-adherent	196	50.6
Current substance use			
Current alcohol	Yes	47	12.1
	No	340	87.9
Current tobacco	Yes	31	8.01
	No	356	91.99
Current khat/caffeinated drinks	Yes	39	10.07
	No	348	89.9
Over all	yes	117	30.2
	No	270	69.8

NB. *Gurage, wolayta, Somaliee, Afar, **Catholic, wakefeta, Hawariyat, ****Farmer, Student

### Psychosocial characteristics of respondents

Regarding to psychosocial characteristics of respondents, majority of them have moderate social support (n = 186, 48.1%) and (n = 142, 36.7%) were stigmatized (Table 2).

**Table 2:** Psychosocial characteristics of respondents among patients with severe mental illness at AMSH, Addis Ababa, Ethiopia, 2019 (n = 387)

Variables	Categories	Frequency	Percent (%)
Social support	Poor	155	40.1
	Moderate	186	48.1
	Strong	46	11.9
Perceived stigma	Stigmatized	142	36.7
	Non-stigmatized	245	63.3

### Quality of life scores of people with severe mental illness

The mean score quality of life in each domain was below 45, as measured in a range from 0-100 using WHOQOL-BRIEF. Nearly half of the respondents scored below the mean score quality of life in each domain. For each domain (mean± SD) it was 41.3±7.5, 42.8±8.2, 38.9±8.9, and 41.8±6.5 for physical, psychological, social and environmental, respectively (Table 3).

**Table 3:** Distribution of quality of life domains among patients with severe mental illness at AMSH, Addis Ababa, Ethiopia, 2019 (n = 387)

Domains	Mean ± SD QOL	Participants who scored below the mean (%)	95% CI
Physical	41.3 ± 7.5	43.9	(40.62, 42.14)
Psychological	42.8 ± 8.2	52.7	(41.94, 43.61)
Social	38.9 ± 8.7	44.7	(38.01, 39.84)
Environmental	41.8 ± 6.5	51.4	(41.12, 42.43)

### Factors associated with quality of life on patients with severe mental illness

The factors associated with quality of life on patients with severe mental illness in the current study are age of the participant strongly positively predicted all of the domains. Social support, the onset of the illness, duration of the illness, comorbid medical conditions, living alone and stigma were the predictors of a lower mean score quality of life in all or at least one domain of quality of life (Table 4).

**Table 4:** Multiple linear regression models of quality of life among people with severe mental illness, Ethiopia, 2019 (n = 387)

Variables	Physical	Psychological	Social	Environmental
	Unstandardized B Coefficient with 95% CI	Unstandardized B Coefficient with 95% CI	Unstandardized B Coefficient with 95% CI	Unstandardized B Coefficient with 95% CI
Age of participants	0.34 (0.27, 0.42)***	0.37 (0.27, 0.47)***	0.53 (0.42, 0.64)***	0.44 (0.36, 0.52)***
Age of onset of illness	0.26 (0.20, 0.33)***	0.27 (0.19, 0.36)***	0.07 (− 0.02, 0.17)	− 0.09 (− 0.07, 0.05)
*Comorbid medical illness*				
No	Ref	Ref	###	###
Yes	− 4.12 (− 5.33, − 2.90)***	− 1.87 (− 3.39, − 0.36)**		
*Stigma*				
No	Ref	Ref	Ref	Ref
Yes	− 2.38 (− 3.38, − 1.37)**	− 1.02 (− 2.33,0.29)	0.74 (− 0.82,2.31)	0.38 (− 0.67, 1.44)
*Residency*				
Urban	###	###	Ref	Ref
Rural			0.93 (− 1.31, 3.11)	− 0.98 (− 2.56, 0.59)
*Living condition*				
With family	Ref	Ref	Ref	Ref
Living alone	− 1.92 (− 3.09, − 0.74)*	0.41 (− 0.92, 1.76)	− 1.27 (− 2.79, 0.24)	− 0.52 (− 1.61,0.57)
*Medication adherence*				
Adherent	Ref	Ref	Ref	Ref
Non-adherent	− 0.42 (− 1.34, 0.49)	0.95 (− 0.24, 2.16)	− 0.59 (− 1.92,0.74)	− 0.17 (− 1.15,0.81)
*Level of social support*				
Strong	Ref	Ref	Ref	Ref
Poor	− 0.65 (− 1.71, 0.41)	− 4.87 (− 6.45, − 3.33)***	− 3.79 (− 5.37, − 2.26)***	− 3.49 (− 4.65, − 2.34)***
Moderate	0.55 (− 0.42, 1.52)	− 1.29 (− 2.74, 0.14)	− 0.30 (− 2.07, 1.47)	− 0.25 (− 1.21,0.71)
*Current sub/user*				
No	Ref	Ref	Ref	Ref
Yes	− 1.19 (− 2.43, 0.05)	− 0.11 (− 1.56, 1.34)	− 1.29 (− 2.87,0.28)	0.79 (− 0.38, 1.96)
*Follow up duration*				
Duration < 5 years	Ref	Ref	Ref	Ref
Duration 5–10 years	0.02 (− 1.06, 1.07)	0.01 (− 1.32, 1.34)	− 3.09 (− 4.61, − 1.58)*	− 2.29 (− 3.42, − 1.13)***
Duration > 10 years	− 1.67 (− 2.89, − 0.41)***	− 0.73 (− 2.28,0.81)	− 0.46 (− 2.24,1.29)	− 0.58 (− 1.83, 0.67)
*Occupation*				
Employed				Ref
Jobless	###	###	###	− 0.68 (− 2.04,0.66)
Private business				0.09 (− 1.31,1.47)
Student				1.22 (− 0.56, 3.02)

*Ref* reference

*p < 0.05, **p < 0.01, ***p < 0.001

^###^ Variable not included in the regression models

## Discussion

The quality of life of people with severe mental illness in Ethiopia was low. The mean scores of quality of life were 41.3, (40.62, 42.14), 42.8, (41.94, 43.61), 38.9, (38.01, 39.84), and 41.8 (41.12, 42.43) with 95% CI for physical psychological, social and environmental domains, respectively. These results are supported by the conclusions of previous studies done in Brazil [24], South Africa [25] and Germany [26].

In contrast to this finding, studies from China and Turkey reported lower quality of life for people with severe mental illness [27, 28]. This could be due to the fact that the studies used a small sample size (China n = 105 and Turkey n = 100) and also the study inclusion of first-visit patients. Moreover, there is good social cohesion and collectivist life style in Ethiopia, not individualistic like developed and western nations. However, studies from Italy, Spain and the UK revealed a higher QOL scores in those countries [27–29]. This might be due to the fact that there were better availabilities of strong health care system and shorter duration of the illness in western nations.

In the present study, it was found that there was a significant correlation between the age of the patients and all domains of QOL. This finding is in line with the studies from Canada, UK and three European countries (France, Germany and UK); as the age of respondents become older, there were a better the quality of life of people with severe mental illness. This might be due to the fact that people mostly accept themselves and their lives as they become older [30–32].

Early onset and longer duration of the illness had a markedly negative impact on the participant’s physical and psychological health of QOL. This finding is parallel with previous researches done in China and Malaysia [33]. The possible explanation might be patients with early onset of mental illness were more likely to have an unfavorable prognosis, higher rates of chronicity, and reduced QOL [34]. Co-morbid medical conditions had also inversely correlated with QOL scores in the physical and psychological domains. This result is congruent with studies done in Sudan and Nigeria [35, 36].

In the current study, physical domain of quality of life of participants who were stigmatized because of their illness was decreased by 2.38 units. This result is supported by studies conducted in Nigeria (51), Jordan [37, 38], Czech [39], and Taiwan [40] as people with the stigma of mental illness perceive themselves as having a lowered self-esteem and self-efficacy which results in less satisfaction in the important life of domain.

Similarly, the better social support of people with severe mental illness positively predicts for higher QOL in almost all domains. This finding is supported by other studies done in Thailand [41]. Germany [19], United state [42, 43], and China [44].

Lastly, participants who were living alone related to a lower physical domain of QOL score as compared with those who were living with the family. The physical health of the participants was reduced by 1.92 units. This finding is in agreement with the study from Sweden [45]. Family support is playing a key role in determining the physical domain of quality of life of severe mental illness [46].

In general, this research found an impaired quality of life of patients with severe mental illness and provides significant clinical and social implications for enhancing the quality of life of patients. As a result, it was suggested that professionals who provide services to patients with severe mental illness improve their quality of life by incorporating the newly innovated positive mental health approach and psychosocial support alongside pharmacological treatment. Health managers and policy makers are also expected to consider this issue in their plan on how to develop strategies for community support programs to increase and enhance social relationships, develop ways to improve public awareness and education to prevent stigma, and encourage psychosocial treatments.

### Limitations

The cross sectional nature of the study design might not show the cause and effect relationship between the outcome and predictor variables. Moreover, the absence of the control group in the current study might not show the real effect of severe mental illness on quality of life of the participants.

### Conclusions and Future directions

Nearly half of the study participants scored below the mean score of the WHOQOL-BRIEF quality of life for all domains. Age of the participant strongly positively predicted all of the domains. Social support, the onset of the illness, duration of the illness, comorbid medical conditions, living alone and stigma were the predictors of a lower mean score quality of life in all or at least one domain of quality of life. This demonstrates a need for improving the quality of life of people with severe mental illness by integration of a positive mental health approach and bio-psychosocial view with biological treatment of severe mental illness. Moreover, strengthen social support, early identification and management of severe mental illness and prevention of stigma among people with severe mental illness. In Collaboration with medical professionals, people with severe mental illness should screen and managed for any comorbid medical conditions.

## Abbreviations

AMSH: Amanuel Mental Specialized Hospital
BD: Bipolar disorder
BSc: Bachelor of science
CI: Confidence interval
CMD: Common mental disorder
CMHS: College of medicine and health science
DSM: Diagnostic and Statistical Manual of American Psychiatric Association
ETB: Ethiopian Birr
HRQOL: Health related quality of life
MDD: Major depressive disorder
MMAS: Morisky Medication Adherence Scale
MSc: Master of science
OSSS: Oslo Social support Scale
QOL: Quality of life
SD: Standard deviation
SPSS: Statistical package for social science
SQOL: Subjective quality of life
WHOQOL-BREF: World Health Organization Quality of Life Brief Version

## Declarations

### Data availability

All the data included in the manuscript can be accessed from the corresponding author Nebiyu Mengistu upon request through the email address of “nebiyumen@gmail.com”.
